# Paediatric oncologists’ perspectives on Strategic solutions to develop Integrated Cancer Palliative Care: feedback intervention theory as an explanatory Framework

**DOI:** 10.1186/s12904-024-01462-y

**Published:** 2024-05-22

**Authors:** Naveen Salins, Krithika Rao, Anuja Damani, Sean Hughes, Nancy Preston

**Affiliations:** 1 Department of Palliative Medicine and Supportive Care, Kasturba Medical College, Manipal, Manipal Academy of Higher Education, Manipal, Karnataka 576104 India; 2 Division of Health Research, Health Innovation One, Sir John Fisher Drive, Lancaster University, Lancaster, LA1 4AT United Kingdom

**Keywords:** Feedback intervention theory, Integration, Palliative Care, Paediatric Oncology, Solutions

## Abstract

**Background:**

Globally, children with cancer often experience delays in palliative care referral or are infrequently referred. Therefore, we conducted a qualitative study to gain insight from paediatric oncologists into what enables or deters palliative care referral. Strategic solutions to develop integrated palliative care was a critical study theme. In this paper, we have explained and interpreted these strategic solutions through the lens of feedback intervention theory.

**Methodology:**

The study findings were interpreted using Kumar’s six-step approach that enabled systematic evaluation of a theory’s appropriateness and alignment with the researcher’s paradigm, methodology, and study findings. It also explained how theory informed analysis and elucidated challenges or the development of new models. The feedback intervention theory appraises the discrepancy between actual and desired goals and provides feedback to improve it.

**Results:**

Strategic solutions generated from the study findings were coherent with the aspects elucidated in theory, like coping mechanisms, levels of feedback hierarchy, and factors determining the effect of the feedback intervention on performance. Paediatric oncologists suggested integrating palliative care providers in the team innocuously, improving communication between teams, relabelling palliative care as symptom control, and working with a skilled and accessible palliative care team. The paper proposes an infinite loop model developed from the study, which has the potential to foster integrated palliative care through excellent collaboration and continuous feedback.

**Conclusion:**

Applying feedback intervention theory can bridge the gap between actual and desired practice for integrated cancer palliative care in paediatric oncology.

## Introduction

Each year, approximately 300,000 children globally are diagnosed with cancer [1]. It is estimated that 90% of children with cancer live in low and middle-income countries, which comprise 84% of the international burden of childhood cancers [2]. While the success rates of childhood cancer treatment might have reduced the need for palliative care in paediatric oncology [3, 4], it is desperately needed in low and middle-income areas where cure rates are low and cancer-related deaths are high [5, 6]. However, survival prognosis cannot solely determine palliative care needs. Approximately 21.6 million children worldwide need palliative care, and 8.2 million need specialist palliative care [7]. Globally, cancer contributes to 6% of palliative care needs in children [8]. Although many children with cancer need palliative care, only a few receive it [7, 9] partly due to oncologists’ gatekeeping behaviour, as evidenced by the studies conducted in high-income countries [10–13].

A multi-country survey of paediatric oncologists in Latin America [14] and Eurasia [15] showed discordance between views and actual practice concerning the timing of palliative care referral, a significant barrier to integrated care. Delayed palliative care referrals, especially in children with haematological malignancies, are often due to the inability to recognise referral triggers, leading to missed opportunities for integration [16]. High-yield palliative care referral triggers do not necessitate translation into practice, and incorporating these triggers into a screening scale might improve referral [17]. This argument is supported by evidence that suggests screening scales [18–20], referral criteria [21], care algorithms [22], standardising practices [23] and periodic audits [24] have the potential to enhance integrated paediatric palliative care. Three studies from the United States showed that embedding palliative care providers in paediatric oncology settings was feasible, acceptable, and improved child and family outcomes [25–27]. The presence of a palliative care team enabled paediatric oncology residents to acquire palliative care skills through imbibed learning [28].

We [29] explored paediatric oncologists’ views on facilitators and barriers to palliative care in a low-middle-income setting. Most findings concerning the development of integrated palliative care in paediatric oncology in our qualitative study [29] mirrored the contemporary evidence. However, in addition to elucidating what enables or deters a referral, paediatric oncologists provided strategic solutions to develop integrated cancer palliative care [29]. In this paper, we interpret and present these strategic solutions through the lens of feedback intervention theory [30].

## Methodology

A qualitative study [29] conducted over eighteen months aimed to explore the views of paediatric oncologists and haematologists on the factors that enable or deter the referral of children with advanced cancer to palliative care. The objectives were to know the participant’s perspectives on the scope of palliative care in paediatric oncology and facilitators and barriers to its referral. The study involved 22 oncologists and haematologists who manage children with cancer, recruited from 13 tertiary cancer centres in India. Research data were gathered through individual, in-person, semi-structured qualitative interviews. Braun and Clarke’s Reflexive Thematic Analysis method was used to analyse the data [31]. The reflexive approach utilized in this study leverages the researcher’s subjectivity as a valuable resource during data analysis [31]. It requires a significant level of critical engagement with the dataset, as the researcher actively interprets the data through their scholarly knowledge, socio-cultural view, ideology, and theoretical suppositions [31]. A critical study theme generated during analysis was strategic solutions, in which paediatric oncologists provided perspectives on developing integrated cancer palliative care. We used feedback intervention theory [30] to interpret these potential solutions in this paper. The interview topic guide, participant information and analysis are provided as tables and supplementary files in the qualitative study paper by Salins et al. (2022) [29].

The study findings were interpreted using Kumar’s six-step approach **(**Table 1**)** [32] using a feedback intervention theory [30] as detailed below. The feedback intervention theory used in this study to interpret oncologists’ perspectives on strategic solutions for integrated care had not been tested in a cancer palliative care setting. Although Kumar’s approach was initially developed for interpreting studies on health education research [32], it still provided a structure for using a theory in a context that was not previously tested [30]. The steps describing the interpretation of the study and findings using feedback intervention theory are detailed in the subsequent section [30].

**Table 1:** Kumar’s Six-Step Approach on using Theory to Interpret Study Findings

1. Comprehensive and Critical Evaluation of a Theory
2. Alignment between Researcher’s Paradigm and Theory
3. Interplay between Research Methodology and Theory
4. Relationship between Theory and Units of Analysis
5. Use of Theory in the Research Process and its Implications
6. Challenges, Adaptations, Development and Critique of the Theory

## Results

### Step 1: Comprehensive and critical evaluation of a theory

Engaging a theory comprehensively and critically involves clarifying its terminology and interpretations, considering other theories that were evaluated, understanding why this theory was chosen, examining its application in different contexts, tracing the evolution of the theory over time, and exploring current debates and criticisms surrounding it [32].

Feedback intervention theory (FIT) was developed in 1996 from historical reviews of feedback and a meta-analysis of studies on feedback intervention [30]. While choosing FIT [30], we considered three other theories suitable for discussing the study findings [29], including social control theory, goal setting theory and clinical performance feedback intervention theory. The premise of social control theory [33] was to use a negative feedback loop to control behaviour and improve performance. The goal-setting theory [34] focuses on setting goals to improve performance. Both were found inadequate to explain the study findings [29]. The closest one to FIT [30] was clinical performance feedback intervention theory [35], a recent adaptation of FIT [30]. It was disregarded as the foundation of the theory was to target the suboptimal performers or systems to boost performance using the best clinical parameters [35]. In FIT [30], the social actor appraises the discrepancy between actual and desired goals, evaluates the performance relative to the goals and then provides feedback [30]. The purpose of the appraisal is not limited to whether to continue or discontinue the relationship but also to reduce the discrepancy between actual and desired goals by improving performance through feedback [30]. To improve clinical practice, it is crucial to use appraisal and feedback as quality improvement strategies. These methods help bridge the gap between current practices and desired outcomes [36]. Appraisal and feedback promote behaviour change, improve the performance of healthcare providers and healthcare [37] and have the potential to improve patient care effectively across various clinical settings [36, 38]. Therefore, FIT [30] was appropriate to discuss the study’s [29] findings. This theory [30] is further critiqued in the discussion section.

**Fig. 1:**
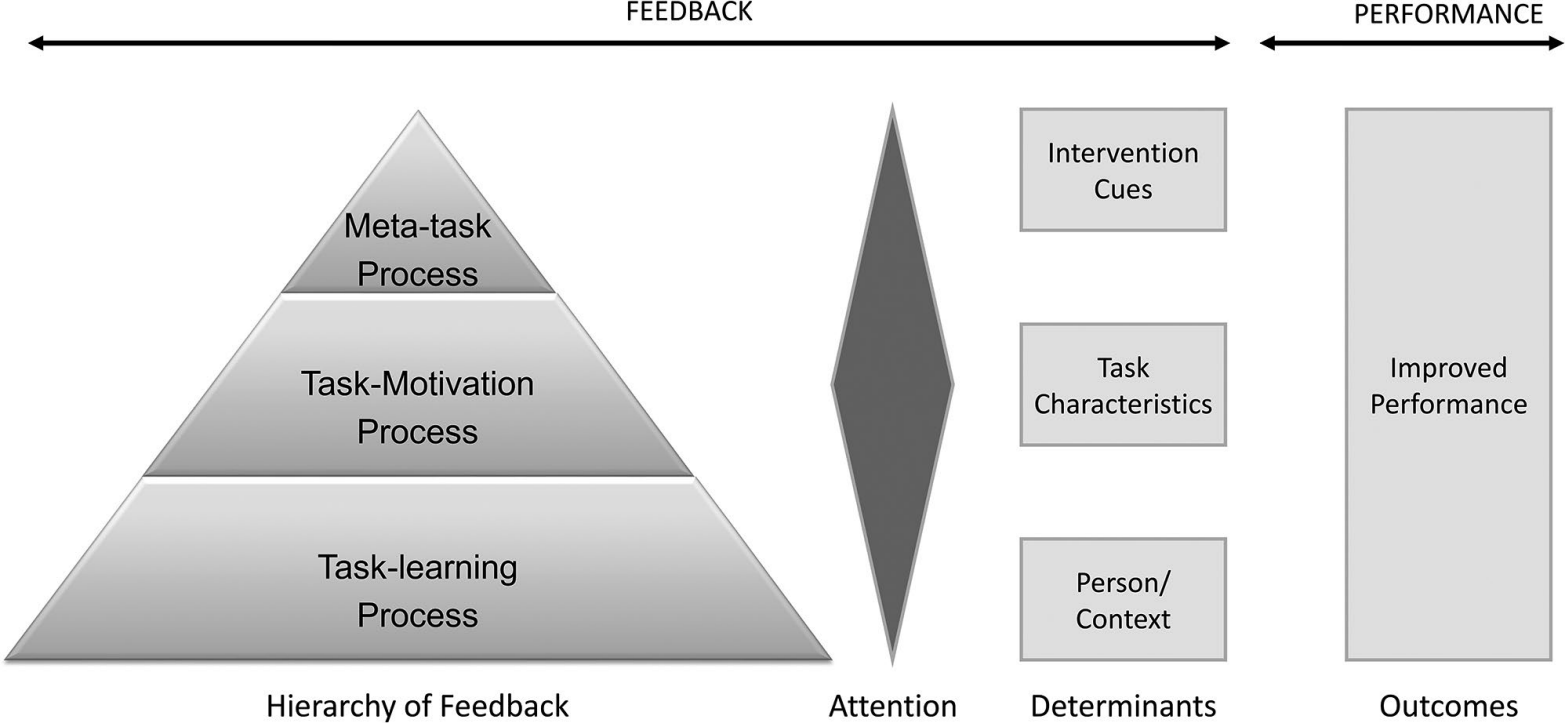
Feedback intervention theory

Feedback intervention theory is detailed in Table 2 and visually represented in Fig. 1. Its application in interpreting the qualitative study [29] findings is described in Step 4.

**Fig. 2:**
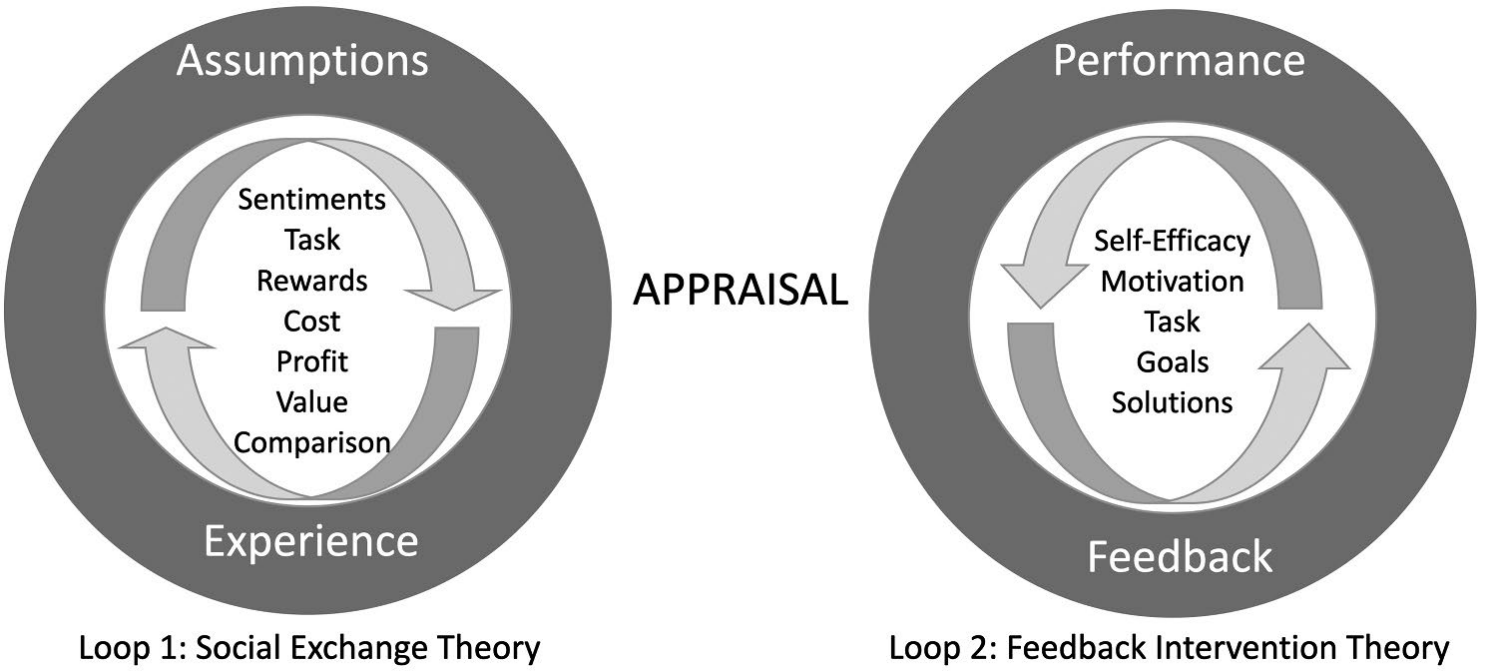
Two theoretical frameworks joining together to form an infinite loop

**Fig. 3:**
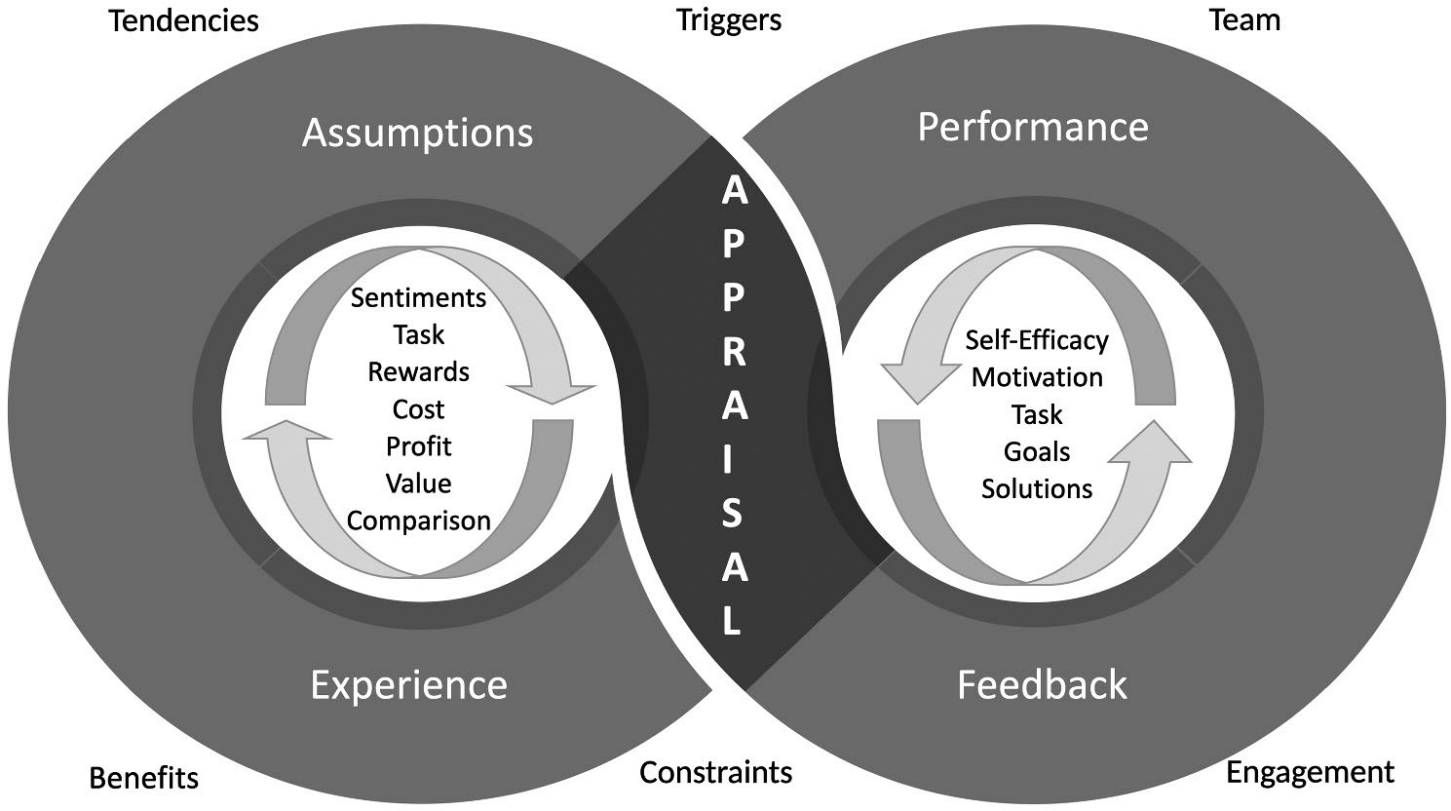
Infinite Loop Model: A proposed aspirational model for integrated palliative care in paediatric oncology

**Table 2:** Concepts of feedback intervention theory

*A. Coping Mechanisms* The feedback intervention theory describes four different coping mechanisms of feedback	A1. Increase Effort: People choosing to increase the effort instead of lowering the standard
	A2. Abandon Standard: Eliminating or disregarding the standard as a coping mechanism.
	A3. Lower Standard: Lowering the standards to meet the desired outcomes
	A4. Reject Feedback: A situation where people refuse to act on feedback
*B. Feedback Hierarchy* Three levels of feedback hierarchy have been described	B1: Meta-Task Process: The meta-task process is at the top of the hierarchy, where the feedback is directed at a personal level or self. It addresses self-efficacy, self-discrepancies, and the emotional response to feedback
	B2: Task-Motivation Process: The middle order is the task motivation process that addresses the focal task processes that stimulate motivation to improve performance
	B3: Task-Learning Process: The task-learning process that deals with the details of the task and the learning process needed to complete the task
*C. Determinants* Three factors determine the effect of the feedback intervention on the performance	C1: Intervention Cues: The first factor is the cues of the intervention. The feedback has to be precise, like a particular task, potential action, or goal to be achieved. It is much better accepted and improves performance when compared to general feedback
	C2: Task Characteristics: The second factor is the nature of the task. The feedback is unlikely to change the performance if the task is highly complex and cognitively demanding
	C3: Person and Context: The third factor is the person receiving the feedback and the situation. The person with functional self-efficacy and self-esteem accepts the feedback better. In a situation where the quantum of change needed to improve performance is less, the feedback is better accepted than in situations demanding drastic changes to improve performance

### Step 2: Alignment between researcher’s paradigm and theory

Here, the researcher identifies their paradigm and explains how the foundations of their worldview align with the theory studied [32].

The primary author of this paper works as a palliative care physician and believes that palliative care is uniquely placed at an intersection of clinical medicine and the humanities [39]. Clinical research should strive to positively impact individuals and society by integrating research with social action to achieve emancipation [40]. Therefore, the transformative paradigm is the research worldview, which is a framework that centres around addressing the inequities of the marginalised and vulnerable communities [41]. Moreover, in the clinical experience of the researcher, children with cancer were infrequently referred to palliative care or late [42]. Oncologists often decide if and when the child has to be referred to palliative care. Vulnerable children suffered needlessly, and families remained ill-informed and were not part of the decision-making process. Their voices were rarely heard. These practices of paediatric oncologists drove the team to conduct this study with the belief that exploring the facilitators and barriers for referral could inform policies and procedures, which might enable more timely access to palliative care.

Evidence suggests that feedback has a positive transformative role in bridging the discrepancy between performance and aspiration [43]. Peer-to-peer feedback involves speaking up when one observes a peer not meeting acceptable standards [44]. It improves performance and quality and is considered a tool for transforming clinical practice [45]. The premise of the FIT is to describe the components of feedback and relate it to performance [30]. In our qualitative study findings [29], the themes on the appraisal of palliative care engagement in terms of facilitators and barriers for referral were followed by strategies to develop integration. As a palliative care physician exploring the phenomenon of palliative care referral, there was a process of co-construction of knowledge where paediatric oncologists and haematologists provided their views and strategies to better palliative care integration to me, the researcher, with palliative care expertise, as feedback.

### Step 3: Interplay between Research Methodology and Theory

It involves explaining how the philosophical foundations of the chosen research methodology interact with the theory used for interpreting study findings [32].

Critical realism serves as a philosophical foundation for research that aims to bring about transformation [46]. It enables the identification of causal generative mechanisms influencing an event and can bring about an emancipatory social change [47]. It is the axiological dimension of research, where the value or moral position assumed by the researcher and study informs decision-making in the research process [48]. My axiological standpoint was identifying the facilitators and barriers to referral that may help mitigate the pain and suffering of children with cancer and their families. The findings of the qualitative study [29] interpreted by FIT [30] were informed by critical realist methodology [46].

A critical realist qualitative multiple case study in a Swiss nursing setting explored feedback on clinical team performance and its transformative role [49]. The critical realist framework helped explain the complexity of the nuanced feedback, its contextual nature and the potential for real-world change [49]. In critical realism, the nature of reality is viewed as layered and placed within social and institutional structures [50]. The knowledge about generative mechanisms changes with context and time [51]. Moreover, there is no linear relationship between the generative mechanisms and the actual outcomes. Therefore, knowing only the empiric aspects may not explain the causal mechanisms [51], with knowledge contextual to the socio-politico-cultural features of a region [52].

Similarly, the feedback process has a robust sociotechnical component [53]. Research has shown that contextual mechanisms play a vital role in explaining why the effects of feedback can vary [54, 55]. Providing feedback can be challenging because it relies on everyone involved having similar expectations, roles, and practices [49]. Additionally, the people giving and receiving feedback may come from different backgrounds and have different levels of autonomy. It can make feedback inconsistent and unpredictable [56]. Furthermore, the context, process, and mechanisms to complete the feedback loop significantly impact outcomes and performance [49].

### Step 4: Relationship between theory and units of analysis

The analysis is divided into two parts. The first part involves analysing the study findings concerning the components of the theoretical framework detailed in Table 1. The second part involves correlating the study results with the emancipatory aspects of the theory, such as gender, power, and sociocultural context [32].

Concerning coping mechanisms described in feedback intervention theory [30], paediatric oncologists preferred to work with a palliative care team with the highest standards. It meant that the palliative care team they would like to work with is regularly available, easily accessible, proactive in seeking referrals, and has dual expertise in oncology and paediatrics.

Three levels of feedback hierarchy have been described [30]. The meta-task process is at the top of the hierarchy, where feedback is directed at a personal level or self. It addresses self-efficacy, self-discrepancies, and the emotional response to feedback. The middle order is the task motivation process that addresses the focal task processes that stimulate motivation to improve performance. The lower order is the task-learning process that deals with the details of the task and the learning process needed to complete the job. For the feedback to be successful, the focus should be on the task motivation process. Feedback focused on the self or the task details is often perceived as negative and less acceptable by the social actor receiving the feedback [30].

Concerning the three levels of feedback explained in the theory [30], the decision to include the palliative care team as part of the oncology service was task motivation feedback [30]. In the study, paediatric oncologists felt that palliative care providers should be introduced as part of the oncology team and be present during initial consultations to encourage collaboration and concurrent care. Additionally, they liked palliative care providers participating in oncology team meetings to ensure the families perceived them at the same level as oncologists. In paediatric oncology, the integration of palliative care is hindered by the lack of concurrent care and advanced care planning (ACP) [57]. To address this issue, paediatric oncologists felt that it might be beneficial to implement a model in which the paediatric palliative care team is situated within the oncology clinic, ward rounds, and meetings and considered a part of the oncology team [58]. While embedding is a good suggestion for an integrated palliative care model [59], it may not be feasible due to global resource constraints in the form of trained staff in paediatric palliative care [60].

One of the task-level [30] feedbacks given was to rebrand the palliative care service and introduce it early as a symptom control team covertly. Other feedback included raising awareness among oncologists about palliative care, improving team communication, and providing palliative care referral guidelines. Oncologists preferred to introduce palliative care early and subtly to reduce stigma and resistance from families. However, negative attitudes towards palliative care among parents often pose a significant obstacle to referrals in paediatric oncology settings [61]. The existing literature on early integration of palliative care in paediatric cancer settings has not yet examined the use of covert relationships as a strategy for achieving early integration [62–65]. One study on nurse-patient relationships revealed how hidden negotiations over time could lead to a mutually beneficial clinical relationship [66]. Another ethnographic study observed how healthcare providers in palliative care settings covertly negotiate their role in decision-making using interpretive repertoires [67].

Adult palliative care providers who consult with children may be met with concerns from paediatric oncologists regarding their medication management, communication, and prognostication skills [68]. It is meta-process or personal feedback [30] by paediatric oncologists to improve patient care quality. While adult palliative care principles can be applied to paediatric care, the two differ significantly [69]. Paediatric palliative care requires a different skill set due to its unique challenges [70]. Adult palliative care physicians can gain the necessary training by working in paediatric palliative care inpatient settings and gaining exposure to the field [70]. Paediatric oncologists recommend adult palliative care providers who work with paediatric patients should receive some training in paediatrics to meet their patients’ needs better.

Three factors determine the effect of the feedback intervention on performance. The first factor was the cues for the intervention [30]. Feedback has to be very specific, like a particular task, potential action, or goals to be achieved. Specific feedback is much better accepted and improves performance than general feedback [30].

The specific feedback from oncologists corresponds to palliative care providers creating a referral pathway, improving awareness of palliative care among oncologists, and participating in oncology team meetings. The oncologists’ feedback included simple and complex tasks related to integrating palliative care services in paediatric oncology. Some complex tasks, such as rebranding the palliative care services as a symptom control team and training providers, require significant effort and time. Shortage of palliative care clinical services [15], as well as a lack of awareness about palliative care among oncologists [71], are also obstacles to integrating palliative care. Providing interprofessional palliative care education to paediatric oncologists has been recognised as essential to improving integration [10, 72]. Improving awareness, communication, and referral guidelines can be achieved with less effort. Additionally, identifying high-yield triggers for palliative care consultation and incorporating them into a screening tool can facilitate early integration [73]. Overall, the feedback suggests that developing an integrated model of palliative care requires a broad range of efforts.

### Step 5: Use of theory in the research process and its implications

It is essential to state if a deductive or inductive approach was used. If the latter was chosen, it’s important to explain how the theory has informed the analysis [32].

The majority of findings of the qualitative study [29] were interpreted using social exchange theory [74]. Social exchange theory explains that people engage in social behaviour to gain or forfeit something of value [74]. In the qualitative study [29], appraisal of palliative care engagement in terms of facilitators and barriers for referral was followed by strategies to foster integration. Social exchange theory [74] was insufficient to discuss the findings of the qualitative study [29] related to strategic solutions, and we felt that the FIT [30] effectively complemented social exchange theory in this respect [74].

Our qualitative study [29] found that paediatric oncologists provided several strategies to facilitate an integrated paediatric palliative oncology model. Being available, proactiveness, adopting a comprehensive approach, embedding palliative care providers in the oncology team, providing concurrent care, inter-team communication, written policies for referral, and improving palliative care awareness amongst the oncologists fostered the partnership between two teams, facilitating a referral. These inductive findings [29] were explained using FIT [30], as detailed in Step 4 above.

### Step 6: Challenges, adaptations, Development and Critique of the theory

This step discussed challenges when using a theory to interpret research findings, adaptations made, and developing new theories or models [32].

As discussed before, the social exchange theory [74] alone was inadequate to discuss the qualitative study’s findings on strategic solutions, and the FIT [30] effectively supplemented the social exchange theory in this regard.

Figure 2 shows the first loop formed by the social exchange theory [74], where previous involvement in the exchange and presuppositions about the provider form assumptions. The social actor appraises the exchange situation for benefits, constraints, tasks, and value. The immediate and long-term experience of the exchange influences assumptions. The assumptions are tendencies that sway the social actor to choose or refuse to participate in the exchange in the presence of triggers.

Figure 2 shows the second loop formed by feedback intervention theory [30], where the social actor appraises the exchange situation and provides feedback to improve performance. Feedback by a social actor is directed at processes that will enhance self-efficacy, motivation, or another social actor’s task learning. The purpose of the feedback is to reduce the discrepancy between the desired and actual goal by improving the collaborating team’s performance and collaboration.

The infinite loop is formed when social exchange theory and feedback intervention theory are joined at the appraisal level. Figure 2 provides a visual representation of two theories before the formation of the infinite loop. The union of two theories at the appraisal level demonstrates the interdependency and influence of these theories on the exchange process. Feedback improves collaboration and performance, which will impact future appraisals. Appraisals determine the experience and assumptions. The assumptions form tendencies, which affect referral behaviour and appraisals in the presence of triggers. The infinite loop model is visually represented in Fig. 3.

### Infinite Loop Model

The infinite loop model is a proposed novel model of integrated care generated from the findings of the qualitative study [29], and union of social exchange theory [74] and feedback intervention theory [30]. The concept of an infinite loop is derived from computer programming, where a sequence of commands makes a loop infinite, and the computer program runs endlessly unless an external intervention terminates the command sequence [75]. In a computer, these non-terminating programs caused by an infinite loop often consume resources without output. However, the infinite loop in the context of integrated palliative care may be aspirational and advantageous in creating a self-activating system for oncology and palliative care to collaborate and improve palliative care access and outcomes.

## Discussion

In one of the earlier definitions, integrated care is described as bringing together inputs, delivery and services management [76]. Inputs in integrated care have a wide-ranging role, from transferring patient information individually to developing a patient navigation system [77]. Some inputs are directed towards professionals and aim to change healthcare providers’ attitudes [77]. Feedback provided by the oncologists and haematologists in this study is a form of professional input to develop the working relationship with the palliative care team. Oncologists feel that there should be a more robust integration between palliative care and oncology [78]. A professional network between two groups based on personal relationships, trust and shared values facilitates integration [79]. Participation of palliative care providers in multi-disciplinary cancer meetings and seamless care coordination between the two teams can facilitate professional networking and integration [80–82]. However, the inputs provided in these meetings are often regarding a patient’s clinical condition [82]. Professional input to improve palliative care providers’ attitudes and performance is seldom offered [77]. Therefore, palliative care providers may remain within their silos, oblivious to the needs and expectations of oncologists and haematologists.

The infinite loop model shown in **Fig. 3** aims to bridge a crucial missing link in the integrated model of palliative care, hypothesising that oncologists’ continuous feedback might facilitate the development of the palliative care team and vice-versa, thereby influencing appraisal. Appraisal during the referral process creates experiences, and these experiences influence assumptions. Assumptions are the tendencies that influence referral behaviour. However, a lack of change in performance following feedback, limited or non-availability of services, and strong presuppositions of the oncologists could interrupt the infinite loop. Ideally, the feedback process should be bidirectional, where palliative care providers receive and give the oncologists feedback. The power differentials between oncology and palliative care teams might hinder bidirectional feedback [51]. Furthermore, we do not suggest that the palliative care team simply accept and acquiesce to this hierarchical system.

A positive referral experience could influence the presuppositions of oncologists and haematologists, facilitating future referrals. An integrated palliative care model is currently understood as effective collaboration [76–78]. Collaboration alongside continuous feedback directed at the palliative care providers could facilitate development and foster integration [77]. This new awareness could inform clinical practice and has the potential to be part of the integrated palliative care approach. Therefore, the infinite loop model is proposed as an aspirational model. Future testing is required to ascertain its role in integrated palliative care.

### Limitations

FIT [30] focuses mainly on individual performance rather than how different systems work together. When developing palliative care, there are many factors to consider, such as education, policies, access, and having adequately trained staff. These are things that the theory may not fully consider. Additionally, the specific impact of feedback and how it works in real-world situations is unclear, which may limit the theory’s applicability. Finally, FIT [30] is seldom tested in a palliative care setting, so its utility is yet to be established.

## Conclusion

Appraisal and feedback can improve integrated cancer palliative care. Applying FIT can bridge the gap between actual and desired goals, ultimately enhancing patient care. In this study, FIT was found useful to discuss and interpret the strategic solutions provided by paediatric oncologists. Incorporating palliative care providers as part of the paediatric oncology team and introducing them innocuously to patients and their families, inter-team communication, and rebranding palliative care as symptom control services were some of the specific feedback provided by paediatric oncologists to support integration. Moreover, they preferred working with a skilled, effective, accessible palliative care team. The infinite loop model developed from this study represents an extended framework where collaboration alongside continuous feedback might further foster integrated palliative care. The present study has unearthed novel insights that could potentially mitigate the existing gaps in the referral of palliative care in paediatric oncology settings. The findings hold promise for facilitating collaboration between paediatric oncology and paediatric palliative care and can pave the way for devising effective strategies for the same.

## Data Availability

(ADM) The data produced or examined during this research has been incorporated into the published article.
