# Correction: Paediatric oncologists’ perspectives on Strategic solutions to develop Integrated Cancer Palliative Care: feedback intervention theory as an explanatory Framework

**DOI:** 10.1186/s12904-024-01471-x

**Published:** 2024-05-28

**Authors:** Naveen Salins, Krithika Rao, Anuja Damani, Sean Hughes, Nancy Preston

**Affiliations:** 1 Department of Palliative Medicine and Supportive Care, Kasturba Medical College, Manipal, Manipal Academy of Higher Education, Manipal, Karnataka 576104 India; 2 Division of Health Researchn, Health Innovation One, Sir John Fisher Drive, Lancaster University, Lancaster, LA1 4AT United Kingdom

**Correction** **:** **BMC Palliat Care 23, 130 (2024)**

**https://doi.org/10.1186/s12904-024-01462-y**

Following publication of the original article [1], the authors reported an error in Figure 1.“Task Motivation “ is printed twice in error. The bottom tier should be “Task-Learning Process “. The correct figure is shown below.

**Fig. 1:**
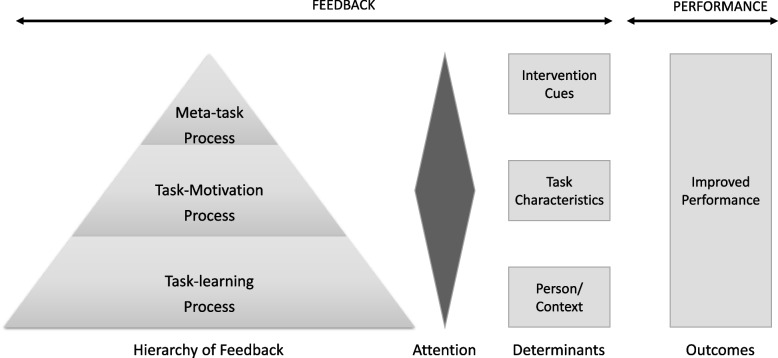
Feedback Intervention Theory

The original article has been updated.
